# Functional analyses of splice site variants in *TCF12*

**DOI:** 10.1186/s40246-025-00758-1

**Published:** 2025-04-26

**Authors:** Angela Borst, Tilmann Schweitzer, Denise Horn, Erdmute Kunstmann, Eva-Maria König, Natalie Pluta, Eva Klopocki

**Affiliations:** 1 Institute for Human Genetics, Biocenter, Julius-Maximilians-University, 97074 Würzburg, Germany; 2 Department of Pediatric Neurosurgery, University Hospital of Würzburg, Würzburg, Germany; 3 Institute for Medical and Human Genetics Charité Universitätsmedizin Berlin, Berlin, Germany

**Keywords:** Craniosynostosis, TCF12, Minigene splice assay, Splicing

## Abstract

Pre-mRNA splicing is a fundamental step in protein synthesis within a cell. Malfunctions during this process can lead to dysfunctional proteins and thus, to a variety of different human diseases. Mis-splicing can be caused by genetic variants influencing many different molecular processes, e.g. splice donor and splice acceptor site variants. Today, the consequences of these variants can be calculated via different in-silico programs. Due to the complexity of the splicing process, however, these predictions are not always correct and should not be used as stand-alone criteria for the classification of potentially disease-causing variants. Therefore, in case RNA from an appropriate tissue is not available additional in-vitro studies, such as a minigene splice assay, which allows functional analyses of potentially disease-causing variants, are necessary to demonstrate an effect on splicing. One example of a human developmental disorder occasionally caused by mis-splicing of transcripts is craniosynostosis. This congenital disorder is defined by the premature fusion of one or multiple cranial sutures in the neurocranium. To date, numerous mutation types in more than 50 genes which are involved in a broad range of different cellular functions and pathways have been associated with craniosynostosis. For instance, the *TCF12* gene encoding the bHLH (basic helix-loop-helix) protein TCF12 (transcription factor 12) is linked to Craniosynostosis 3 (OMIM: 615314) which exhibits a Saethre-Chotzen (OMIM:101400) like phenotype. In this study, we report a pipeline for functional validation of potential splice site altering variants. First, we describe the identification of two novel genetic variants and revalidation of one previously described genetic variant in patients with craniosynostosis. According to in-silico predictions, the splicing of the corresponding transcripts is altered, and the variants are potentially disease causing. We subsequently classify the consequences of alterations in *TCF12* experimentally. The suspected aberrant splicing was investigated via an in-vitro minigene splice assay. In two out of three variants, the in-silico prediction and in-vitro experiments were consistent. In all variants a significantly reduced transcriptional activity was demonstrated. In summary, the combination of in-silico prediction and functional assays allowed us to classify the variants as likely pathogenic without the need for additional patient material.

**Supplementary Information:**

The online version contains supplementary material available at 10.1186/s40246-025-00758-1.

## Introduction

Splicing of pre-mRNA is an important step in generating a functional protein from the respective human gene. During this process the noncoding introns between the coding exons are removed [1–3]. The splicing process is performed by the spliceosome, a protein-RNA complex with essentially two functions: [1] the recognition of the specific sequences of the boundaries between the exons and the introns and the final removal of the introns and [2] the reconnection of the spliced exons [1, 4]. Next to the canonical splice site sequences, including the splice acceptor, splice donor and the branch point, also cis-elements, including exonic and intronic splice enhancers and silencers, are important for the appropriate splicing of pre-mRNAs [1–4]. Variants located at the canonical splice site influence the ability of the spliceosome to recognize the exon-intron boundaries and can result in exon skipping [1, 4, 5]. In addition, it can result in partial loss of the exonic sequence or partial inclusion of the intronic sequence into the spliced mRNA [1, 4, 5]. In most cases the underlying genetic cause is the substitution of one nucleotide [4]. Pathogenic splice site variants have been described for many different genes that are associated with various diseases [1, 4]. One congenital disorder we are interested in is craniosynostosis which has also been demonstrated to result from pathogenic genetic variants influencing splicing [6]. Clinically craniosynostosis is caused by the premature fusion of one or more cranial sutures [7, 8]. The cranial sutures are essential for the growth of the skull, the related growth of the brain and the deformability of the skull. Proper development and growth of the skull requires the interaction of many different signaling pathways, with complex receptor-ligand interactions. Defects in proteins of this complex network caused by congenital variants can lead to craniosynostosis [7, 8]. The incidence of craniosynostosis is estimated to be between 1 in 1,800 and 1 in 2,400 newborns [9, 10]. Depending on the suture exhibiting the premature fusion and the resulting skull shape due to compensatory growth of the skull parallel to the fused sutures at the unaffected sutures [11–14], craniosynostosis can be classified clinically. Synostosis of the sagittal suture is thereby the most common type [11, 13, 15]. Due to the inhibited cranial growth, several secondary effects and clinical complications can be observed in patients with craniosynostosis, such as increased intracranial pressure (ICP) and intellectual disabilities or facial deformations [11, 16–19]. A further classification of craniosynostosis can be made between nonsyndromic and syndromic forms, with nonsyndromic forms being the most observed manifestation, accounting for 70% of all craniosynostosis diagnoses [11, 20]. Apart from the nonsyndromic form to date at least 150 syndromes are associated with craniosynostosis which has a large variety of genetic causes [20]. Different chromosomal abnormalities including deletions and duplications, which are located on almost all chromosomes, are described to be disease-causing [11, 20, 21]. In addition, pathogenic variants in at least 57 genes, including both gain-of-function (GOF) and loss-of-function (LOF) variants, are associated with craniosynostosis [11, 22].

These genes encode proteins involved in a variety of different signaling pathways, one of them being the Fibroblast growth factor (FGF) signaling pathway. A more recently identified gene linked to craniosynostosis is *TCF12* encoding the bHLH (basic helix-loop-helix) protein TCF12 (transcription factor 12; also called HEB) which belongs to class I proteins, also called E proteins [6, 23, 24]. Different *TCF12* transcripts, which are spliced into two isoforms, including or lacking exon 15, have been identified. Furthermore, an isoform utilizing an alternative start codon is known, lacking exons 1 to 8 but including exon 9a, which encodes the Alt domain [6, 25]. In addition to the bHLH domain important for DNA binding, TCF12 contains two activation domains (AD1/AD2) and a Rep domain. LOF variants in the *TCF12* gene are associated with Craniosynostosis 3 (OMIM: 615314), which shows a Saethre-Chotzen (OMIM:101400) like phenotype [6]. The craniosynostosis-causing pathogenic variants described in *TCF12* are mainly located either in or near the bHLH domain and result in a frameshift and/or premature stop codon [6, 26]. Intragenic as well as complete *TCF12* deletions have also been reported as a cause of craniosynostosis [27–29]. Foss-Skiftesvik et al. present a systematic review on pathogenic *TCF12* [30].

The spectrum of genes with potential splice site variants is broad. We expect many yet undiscovered and potentially disease-causing variants and even previously described variants that are missing functional classification and experimental investigations. This is often due to the lack of patient RNA or patient-derived primary cells obtained from the appropriate tissue of the patient which is needed for a proper interpretation of variants’ consequences on transcript splicing and finally the resulting protein [4]. With our combination of in-silico prediction, in-vitro mutation-matched minigene splice assay and Luciferase assays, we analyzed potential splice site variants, detected by Exome sequencing, in a fast and reliable way. This allows us to reveal the impact of a variant on the mRNA transcript and to make more accurate predictions about the effect on the translated protein and its function.

## Materials and methods

### Sample enrichment, NGS and bioinformatic analysis

For the genetic analysis of patient genotypes with next-generation sequencing (NGS), gDNA from blood samples was prepared. The Nextera XT DNA Library Kit (Illumina) was used for sample enrichment and the xGen Exome Research Panel (IDT) was used for subsequent probe hybridization. Exome sequencing was performed on the Next Seq 500 (Illumina).

The generated data were analyzed via GenSearch NGS software (Phenosystems) via an in-silico craniosynostosis gene panel (see Table S2) and the interactive biosoftware, Alamut Visual (created by Sophia Genetics SA, v.2.15.0). Variants were filtered in GenSearch NGS according to their quality (frequency > 0.15, coverage ≥ 10x), minor allele frequency (MAF < 0.02) and position (± 20 bp into the intron). The detected variants were classified considering amino acid changes and their predicted impact on the protein (PolyPhen2; Mutation Taster; SIFT), possible changes in the splice site (SSF, MaxEnt, NNSPLICE, and Gene Splicer) and their presence in the population (gnomAD v2.1.1) according to ACMG guidelines [31].

### Ethics request

Written informed consent was obtained from each subject. The study was approved by the Institutional Review Board of the University Hospital Würzburg (Votum Nr. 89/13).

### Minigene splice assay

**Fig. 1:**
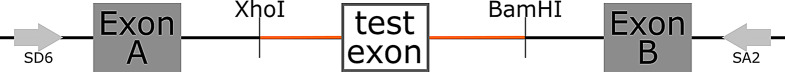
Schematic illustration of the cloned variant matching the pSPL3b-cam vector. Schematic representation of the pSPL3b-cam vector showing the exon including the variant to be tested (test exon) and flanking intronic regions (in orange) of the gene under investigation. The restriction sites XhoI and BamHI used for cloning are shown. The flanking artificial exons A and B (in gray) present on the vector and the sequencing primers SA2 and SD6 are indicated

For the minigene splice assay, mutation-matched pSPL3b-cam plasmids were generated to investigate the effects of the detected variants on splicing. For this purpose, the inserts were amplified with specific primers (see Table S1) from wild-type and patient DNA using Q5 High-Fidelity DNA Polymerase (New England Biolabs). For each patient, the inserts contained the respective exon affected by the variant, and approximately 500 bp of the flanking intronic sequences. The inserts were cloned between the BamHI and XhoI (New England Biolabs) restriction sites of the pSPL3b-cam [32] vector using the T4 DNA Ligase (New England Biolabs) (see Fig. 1).

U-2 OS cells were grown in Dulbecco’s modified Eagle medium (DMEM, Thermo Fischer Scientific) supplemented with 10% fetal calf serum (FCS, Anprotec) at 37 °C and 5% CO_2_. For the minigene splice assay, 2 × 10^5^ U-2 OS cells were grown in 6-well plates (Greiner Bio-One GmbH) with cell culture medium (DMEM + 10% FCS) to a confluence of 70–80%. The cells were subsequently transfected with 2 µg of either the control (plasmid without an insert), the wild-type, or patient-specific insert carrying pSPL3b-cam plasmid with 6 µl of FuGene 6HD (Promega). 24 h after transfection, cells were harvested with Trypsin (Sigma Aldrich) and RNA was isolated from the cells via the RNeasy^®^ Kit (Qiagen). Subsequently cDNA was synthesized with the FIREScript^®^ RT cDNA synthesis kit (Solis Biodyne).

### PCR and Sanger sequencing

The splicing of the pSPL3b-cam plasmid without inserts (control) or with either the wild-type or the patient-specific insert was analyzed via PCR using the SD6 and SA2 primers (see Supplementary Table 1) and PlatinumTaq-DNA-Polymerase (Invitrogen Thermo Scientific) with a standard PCR program. The PCR products were investigated on a 1% agarose gel with Hyper Ladder™ 1 kb (Bioline GmbH) as a marker. In addition, the splice products were analyzed via Sanger sequencing using the BigDye™ Terminator v1.1 Cycle sequencing Kit (Applied Biosystems) and the ABI 3230 × 1 Genetic Analyzer (Applied Biosystems).

### Luciferase assay

For the Luciferase assay different plasmids were used. The PIS14 plasmid (Emmanuelle Huillard; Sorbonne Université, Paris) either with a *TCF12* cDNA (NM_207037.2) insert or without an insert, was used as a control. The *TCF12* insert included the coding region with either the wild-type sequence or the aberrant one calculated from the minigene splice assay. The plasmids containing the aberrant *TCF12* sequences were generated via the Q5 Mutagenesis Kit (New England Biolabs) according to the manufacturer’s protocol using mutation specific primer pairs (see Supplementary Table 1). Additionally, the PGL4.27 plasmid (Promega) containing an Ebox-motif (sequence: CACCTG) was used for the firefly luciferase expression, which depends on the transfected overexpressed *TCF12.* Further, the pGL4.74 (Promega) for the Renilla expression was used as a transfection control. HEK293T cells were grown in DMEM supplemented with 10% FCSat 37° C and 5% CO_2_. For the Luciferase assay, 1.5 × 10^5^ HEK293T cells were grown in cell culture medium (DMEM + 10% FCS) in 12-well plates (Greiner Bio-One GmbH) until they reached 70 to 80% confluence. The cells were subsequently transfected with 2 µg of plasmid DNA by using FuGene HD (Promega). After 24 h the cells were harvested using the Passive Lysis buffer (Dual Glow Luciferase Reporter Assay Kit; Promega) and the cleared lysate was transferred to a 96-well plate (Greiner Bio-One GmbH). For the subsequent Luciferase assay measurement, the Dual Glow Luciferase Reporter Assay Kit (Promega) and a plate reader (Mithras) were used. The relative Luciferase activity and significance were calculated via Excel (Microsoft).

## Results

By exome sequencing we identified one previously described and two yet undescribed heterozygous variants in *TCF12* (NM_207037.2) (see Table 1). A possible change in splicing was indicated by in-silico splice prediction tools for all three variants.

**Table 1:** Identified genetic variants and affected sutures

Term	Gene	Transcript ID	Variant	Mutation type	Affected suture
P1	*TCF12*	NM_207037.2	c.1467 + 1G > C	splice site	coronal
P2	*TCF12*	NM_207037.2	c.1468–7 A > G	splice site	coronal
P3	*TCF12*	NM_207037.2	c.1746-5_1746-1dup	splice site	coronal

**Fig. 2:**
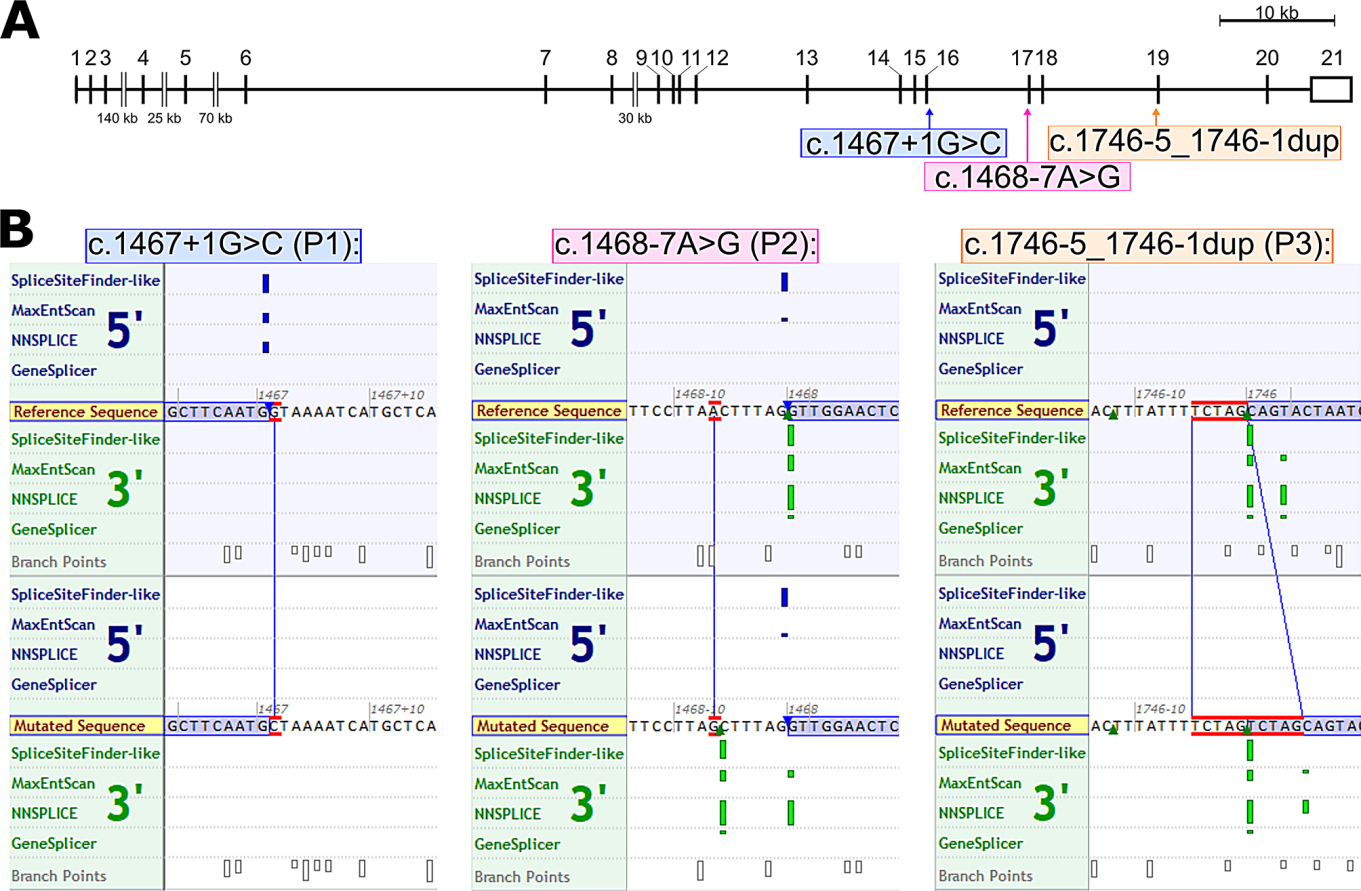
Localization of the variants in the *TCF12* gene and their splice predictions. **A**: Genomic structure of *TCF12* and the positions of the three variants. **B**: Splice prediction of variants P1, P2 and P3. P1: loss of the splice donor site in the lower panel (blue bars); P2: gain of the cryptic splice acceptor site and downranking of the initial splice acceptor site in the lower panel (green bars); P3: shift of the splice acceptor site in the lower panel (green bars)

Patient 1 (P1) is a female showing a bilateral coronal synostosis. Genetic analysis of P1 via NGS and the craniosynostosis gene panel, revealed a heterozygous variant in exon 16 of *TCF12* (c.1467 + 1G > C, see Fig. 2A; Supplementary Fig. 1A). Segregation analysis of the unaffected parents revealed that the mother carried the same variant as her daughter but did not show any obvious craniosynostosis phenotype (see Supplementary Fig. 1A). Patient 2 (P2) is a female with coronal synostosis. We detected a previously described [33] heterozygous variant in intron 16 of the *TCF12* gene (c.1468–7 A > G, see Fig. 2A; Supplementary Fig. 1B). The DNA of the asymptomatic parents was not available for testing. Patient 3 (P3) is a female with coronal synostosis. We detected an undescribed heterozygous variant in intron 18 of the *TCF12* gene (c.1746-5_1746-1dup, see Fig. 2A; Supplementary Fig. 1C). Segregation analysis of the unaffected parents showed that the mother of P3 is carrier of this *TCF12* variant. The two sisters of P3, who were clinically unaffected were not genetically tested (see Supplementary Fig. 1C).

### Splice analysis

In-silico splice predictions and in-vitro minigene splice assays were performed with all three variants to predict splicing of the affected genes in-silico and to confirm the prediction experimentally. The localization and splice prediction of the variants are shown in Fig. 2B. For P1 the splice prediction indicated loss of the splice donor of exon 16 (see Fig. 2B) and the associated skipping of exon 16. In case of the variant of P2 the splice prediction tool indicated gain of a splice acceptor site at the position of the variant (indicated by the additional green bars in the lower panel) as well as a downranking of the initial splice acceptor site of *TCF12* exon 17 (see Fig. 2B). For the duplication at the splice acceptor of exon 19 detected in P3 the splice prediction tools indicate the gain of a cryptic splice site located in the duplicated intronic region. Additionally, splice prediction tools indicate the loss of the initial splice site. This would result in the insertion of five base pairs of intron 18 into exon 19 of the spliced *TCF12* mRNA (see Fig. 2B).

**Fig. 3:**
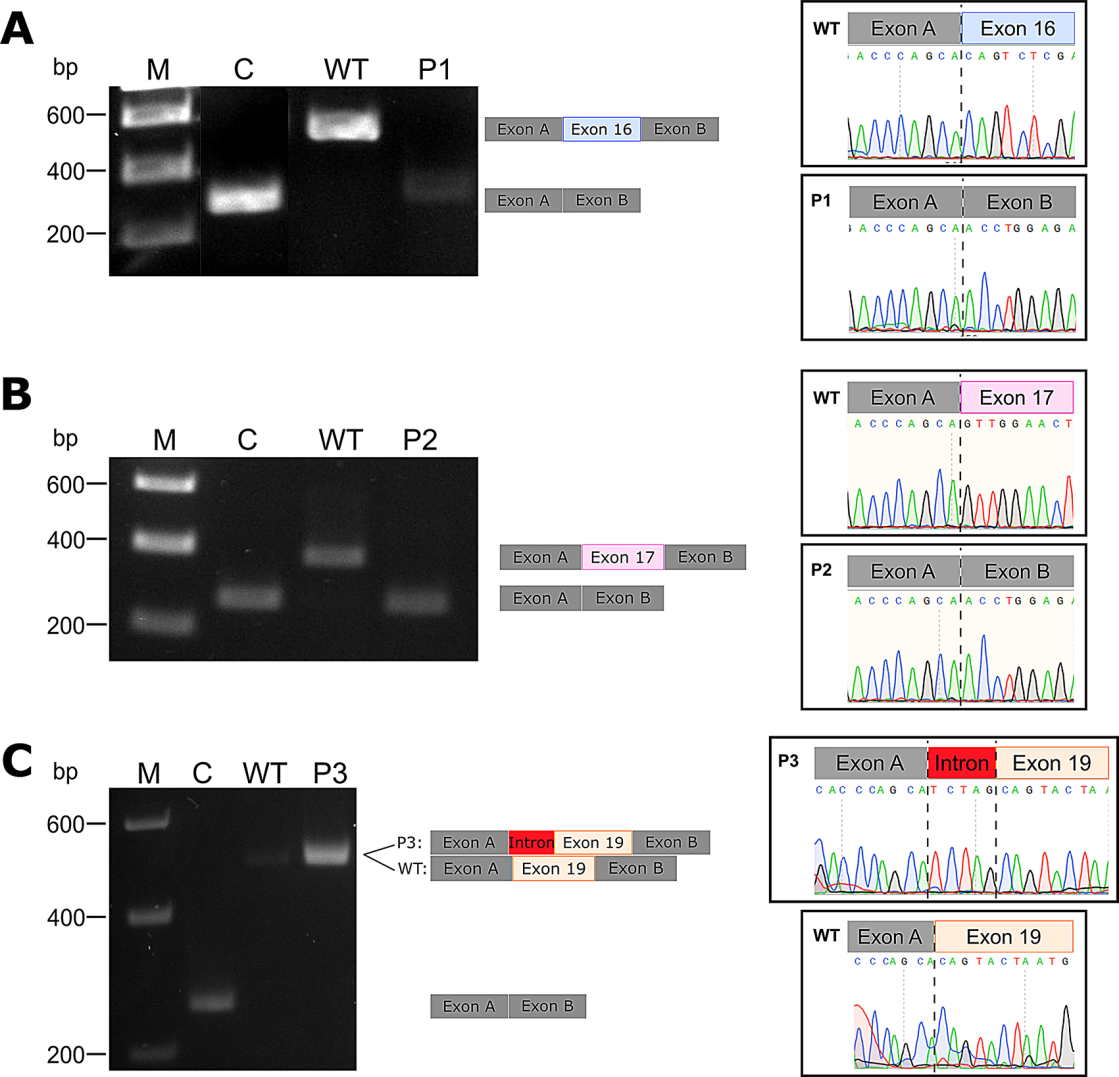
Minigene splice assay of the three *TCF12* variants. **A**: Results of the minigene splice assay for variant P1 by PCR, gel-electrophoresis, and Sanger sequencing. Showing skipping of exon 16 in the splice product. **B**: Results of the minigene splice assay for variant P2 by PCR, gel-electrophoresis, and Sanger sequencing. Showing skipping of exon 17 in the splice product. **C**: Results of the minigene splice assay for variant P3 by PCR, gel-electrophoresis, and Sanger sequencing. The partial inclusion of intron 18 in the splice product is shown

The minigene splice assay was performed with the pSPL3b-cam vector including exon 16 and flanking intronic regions of the *TCF12* gene and the pSPL3b-cam vector without insert as a control was performed with WT and patient DNA (P1). On the agarose gel, the PCR on the cDNA revealed a band with a size of ~ 600 bp for the WT, a band of ~ 300 bp for the control (C), and a band of ~ 300 bp with a lower intensity for the variant. Sanger sequencing confirmed the complete skipping of exon 16 in variant P1 (see Fig. 3A). The PCR on the splice products revealed an ~ 300 bp product for the control and variant P2 as well as a ~ 400 bp product for the wild-type sequence. Sanger sequencing confirmed skipping of exon 17 in the cDNA sequence of the variant construct (see Fig. 3B). The splice products of the minigene splice assay of variant P3 showed no detectable change in the size of the splicing products on the agarose gel. A band of ~ 500 bp in length was visible in both the wild-type and variant P3 sequences. Sanger sequencing revealed the insertion of five base pairs originating from intron 18 into the splice product (see Fig. 3C).

**Fig. 4:**
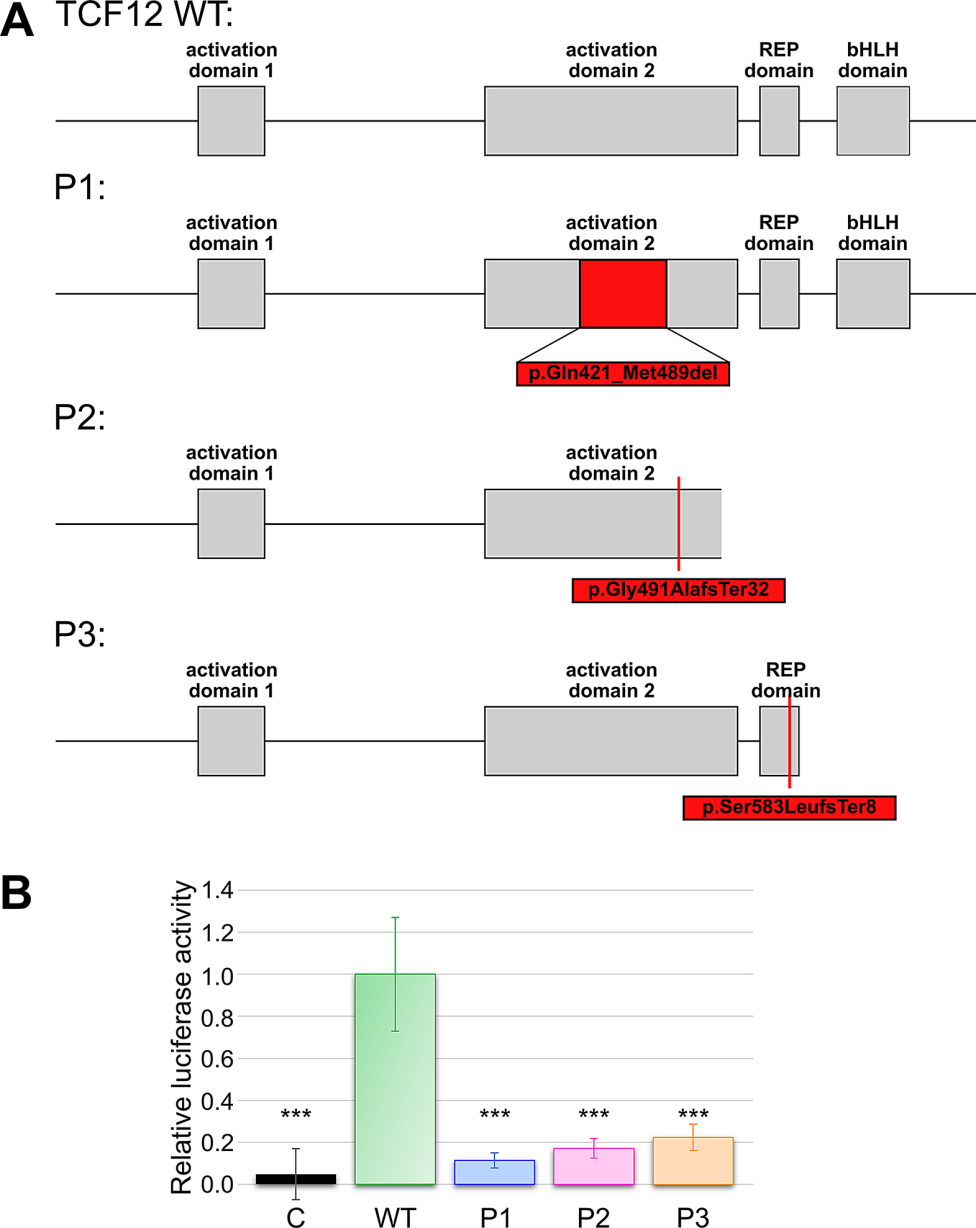
TCF12 protein prediction and functional analysis via a Luciferase assay. **A**: Protein prediction resulting from the aberrant splicing caused by the variants (deletion in dark red, frame shifts in bright red). **B**: Luciferase assay results for the control (**C**), TCF12 wild-type (WT) and aberrant TCF12 proteins. Compared with that of TCF12 WT, highly significant reductions in the transcriptional activity of the P1, P2 and P3 TCF12 proteins were detected. Significance was calculated to TCF12 WT (***: *P* < 0.001)

The c.1467 + 1G > C *TCF12* variant in P1 which was predicted to cause loss of the splice donor of exon 16 was confirmed in-vitro to result in skipping of exon 16 in the splice product. This suggests an in-frame loss of 69 amino acids in the translated TCF12 protein (p.(Gln421_Met489del)). The deleted amino acids are part of the AD2 domain (Fig. 4A). In-silico prediction for the previously described c.1468–7 A > G *TCF12* variant [33] indicated the gain of a cryptic splice acceptor site upstream of the initial splice site. Yoon et al. assumed an effect on splicing on the basis of the generated cryptic splice acceptor but did not perform a functional splicing analysis [33]. One consequence could be partial inclusion of intron 16 in the splice product as previously described for the c.1468-20T > A variant in the *TCF12* gene [6]. However, our minigene assay clearly confirmed the skipping of exon 17, leading to the prediction of a frameshift and premature stop in the TCF12 protein (p.(Gly491AlafsTer32)). This occurs most likely because of a weakening of the initial splice acceptor site of exon 17, which consequently results in the TCF12 protein lacking the Rep and bHLH domains (Fig. 4A). Thus, our in-vitro data clearly show that the predicted gain of a cryptic splice acceptor weakens the initial splice site leading to skipping of the complete exon. In case of the duplication (c.1746-5_1746-1dup) in *TCF12* intron 18 the in-vitro results match the in-silico prediction of aberrant splicing i.e. partial inclusion of intron 18. This modification presumably leads to a frameshift and eventually a premature stop codon in TCF12 (p.(Ser583LeufsTer8)). Consequently, the bHLH domain is lost in the aberrant TCF12 protein (Fig. 4A).

Furthermore, the influence of the variants on the capability of DNA binding to the Ebox-motif and on transcription factor activity was analyzed via a Luciferase assay. Compared with the wild-type TCF12 protein, all three aberrant proteins presented significantly decreased relative luciferase activity, which was comparable to that of the control (C). Notably, the aberrant protein partially lacking the AD2 domain (P1) thereby showed the weakest activity. The two proteins lacking among others the bHLH domain due to premature stop codon (P2, P3) presented greater relative luciferase activity, which was still significantly lower than that of the wild-type TCF12 (see Fig. 4B).

## Discussion

We identified previously undescribed heterozygous *TCF12* variants in two patients with craniosynostosis and a previously described heterozygous variant in a third patient. All variants are located close to canonical splice sites in this known disease-causing gene and are predicted to cause incorrect mRNA splicing. *TCF12* is a known and well-studied gene related to craniosynostosis, and pathogenic splice site variants are already described as potentially disease causing [6, 34].

To date, in the AD2 domain only pathogenic frameshift and/or premature stop variants, which result in the additional loss of the bHLH domain are reported [6]. In the case of variant P1, we observed partial loss of the AD2 domain, without any consequences for the bHLH domain. Previously described craniosynostosis causing pathogenic splice site variants that involve the AD2 domain are thought to cause out-of-frame deletions and premature stop codons which imply additional loss of the bHLH domain [6, 35]. In other E proteins, such as E12 or E2-2, complete deletion of the AD2 domain has a negative effect on the transactivation function of the transcription factor [36, 37]. It is also known that the AD1 and AD2 domains in E proteins are involved in the recruitment of coactivators and corepressors. Since deletion of either AD1, AD2, or the bHLH domain leads to growth degradation in fibroblasts these domains are critical for ideal protein function [38]. Moreover, studies have shown that the helical AD1 and AD2 domains of E2-2 are required for interaction with the KIX domain of the transcriptional coactivator CBP [37, 39]. These data for other E-proteins fit with the results obtained for the functional testing of the TCF12 protein lacking the AD2 domain. We were able to clearly show that the loss of the AD2 domain significantly influences the transcriptional function of the protein. Taken together, the in-frame deletion of 69 amino acids in the AD2 domain, due to aberrant splicing, influences the activity of TCF12 which explains the bilateral craniosynostosis phenotype in P1. Because of the in-vitro functional studies of the presumably resulting aberrant protein we classify the c.1467 + 1G > C variant as likely pathogenic (ACMG guidelines; criteria PS3) in P1.

The predicted TCF12 proteins due to P2 and P3 variants lack the bHLH domain, which is required for dimerization of TCF12 with either itself or other proteins such as TWIST1 [6, 40]. Comparison with previous studies on *TCF12* variants in patients with craniosynostosis revealed an accumulation of missense and frameshift variants in the 3’ region of the protein affecting the bHLH domain [6]. The two missense variants (p.(Gln638Glu) and p.(Leu624Pro)) negatively affected the transactivation activity of TCF12 when co transfected with *TWIST1* in cell culture experiments [6]. Topa et al. previously described c.1769delT, leading to p.(Leu590*), with a premature stop codon at a comparable position as a likely pathogenic variant for craniosynostosis [41]. A microdeletion containing exon 19 and exon 20 has also been previously reported as cause of craniosynostosis [27]. The importance of an intact bHLH domain is clarified by the knowledge of disease-causing variants in the bHLH domain of related transcription factors, such as TCF4 [42]. In addition, variants present in the bHLH domain of TWIST1 have been reported to cause Saethre-Chotzen syndrome [43, 44]. Overall, we were able to show the influence of the P2 and P3 variants on splicing via the minigene splice assay. Additionally, the functional testing of the presumed aberrant proteins showed a decreased function of the transcription factor TCF12. Phenotypically all three patients with *TCF12* variants exhibit coronal synostosis which is typical in *TCF12* related craniosynostosis [6, 26]. Although *de-novo* occurrence of *TCF12* variants could not be proven in P1, P2 and P3, we classified all three variants as likely pathogenic due to the known reduced penetrance of *TCF12* mutations [6, 26].

In summary, we describe two novel and one previously known splice site variants in *TCF12*. We provide experimental evidence of a deleterious effect on protein function caused by incorrect splicing. This allowed us to classify all three *TCF12* variants (P1, P2 and P3) as “likely pathogenic”. These examples demonstrate the necessity of functional in-vitro studies to indicate the pathogenicity of splice variants especially if in-silico predictions indicate a potential effect on splicing. Furthermore, the method pipeline reveals the possibility of functional analysis of potential splice site variants without the need of additional patient derived material. This approach is not only limited to analyze variants at or close to canonical splice sites but also suitable to study deep intronic variants predicted to cause aberrant splicing. An additional challenge in variant classification is the inconclusive segregation analysis of variants in genes with reduced penetrance, which are common in craniosynostosis. Therefore, additional functional analysis of these variants is recommended to provide evidence of pathogenicity and thus, are of great importance in reliable variant classification.

## Electronic supplementary material

Below is the link to the electronic supplementary material.

Supplementary Material 1: NGS results and pedigrees of the three patients. A: Result of NGS and segregation analysis of P1 and clinically unaffected parents. B: Result of NGS of P2. C: Result of NGS and segregation analysis of P3 and clinically unaffected parents. AA: amino acid; NA: not analysed; w/w: wild-type sequence; w/m: heterozygous variant carrier.

Supplementary Material 2: Used primers.

Supplementary Material 3: Gene list of in-silico panel.

## Data Availability

The datasets used and analyzed during the current study are available from the corresponding author on reasonable request.
