# Supplementary figures and images for: Functional analyses of splice site variants in TCF12
Source: Hum Genomics. 2025 Apr 26;19:45. doi: 10.1186/s40246-025-00758-1 (PMC12034182; doi:10.1186/s40246-025-00758-1)

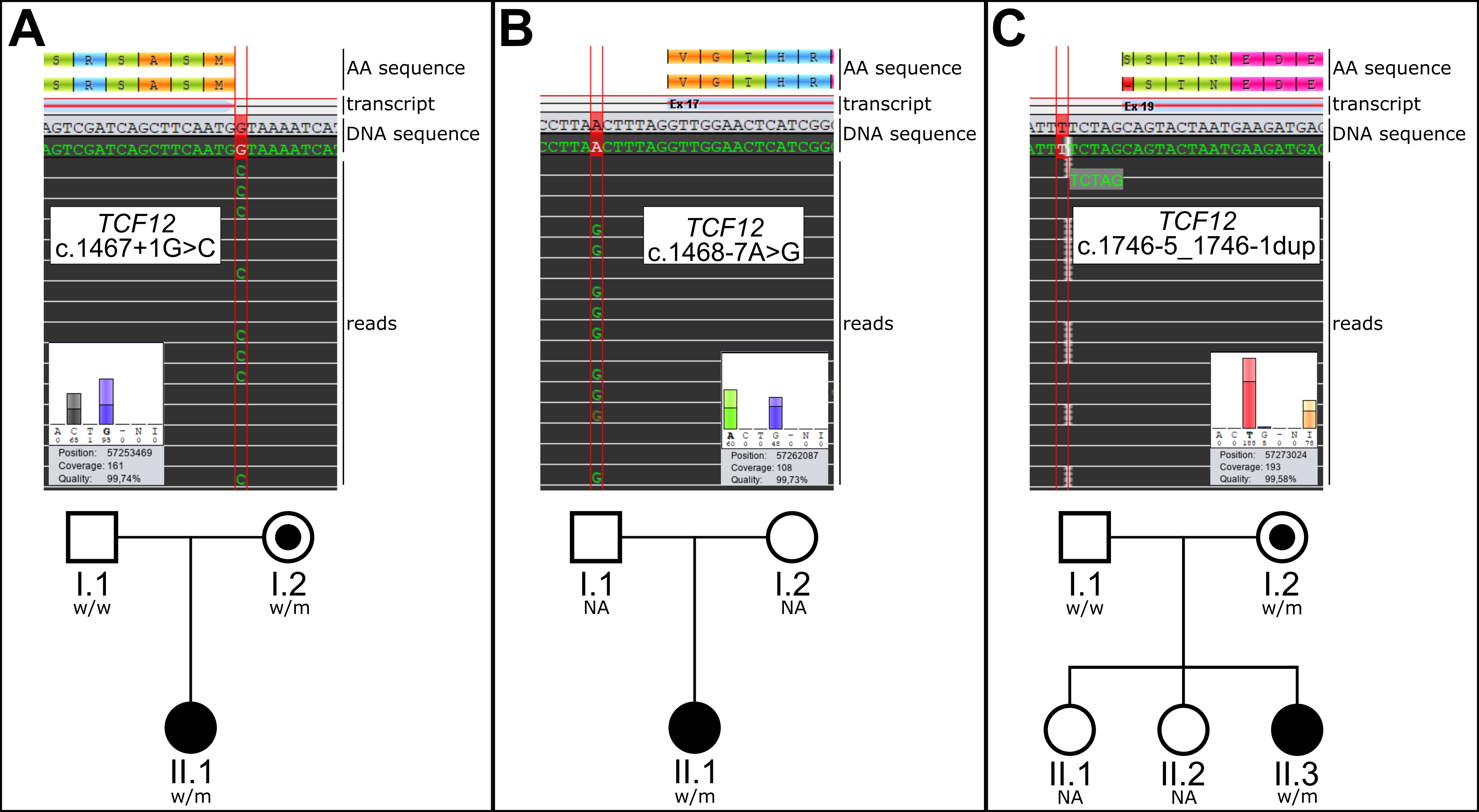

Supplement: Supplementary file 1 — Supplementary Material 1: NGS results and pedigrees of the three patients. A: Result of NGS and segregation analysis of P1 and clinically unaffected parents. B: Result of NGS of P2. C: Result of NGS and segregation analysis of P3 and clinically unaffected parents. AA: amino acid; NA: not analysed; w/w: wild-type sequence; w/m: heterozygous variant carrier. [file 40246_2025_758_MOESM1_ESM.png]
